# Supplementary material for: MAPPinfo ‐ mapping quality of health information: Validation study of an assessment instrument
Source: PLoS One. 2023 Oct 23;18(10):e0290027. doi: 10.1371/journal.pone.0290027 (PMC10593225; doi:10.1371/journal.pone.0290027)
Supplement: Attachment — Submitted filename: Response to Reviewer.docx [file pone.0290027.s001.docx]

Letter of appeal: PONE-D-22-19542

Dear editor, dear Dr Mukhtiar Baig,

we are extremely disappointed that our manuscript on the development of a new instrument measuring the quality of health information has been rejected, not least because the decision to do so has taken three months.

The rejection was all the more frustrating for apparently having been attributable to the comments of only one reviewer.

As young, motivated scientists were involved in preparing the manuscript and they are understandably extremely dismayed at having been fobbed off on the basis that no better reviewers could be found, I feel it is my responsibility to appeal your decision. After all, young scientists profit most from fair and constructive criticism, and I feel it would be a mistake to simply submit our article to alternative journals that are not so well suited to them.

I would like to appeal the decision to reject our paper for publication, on the basis of our opinion that the reviewer’s comments are partly superficial, partly poorly justified and not giving reason for a rejection.

*Reviewer #1: The comments for the manuscript entitled “MAPPinfo - mapping quality of health information: validation study of an assessment instrument” are as follows:*

*1- In the abstract, the background and aim of the study need to be revised, as they do not support the title of the paper.*

**Response:** We do not understand in which regard background and aim not “support” the title. If the meaning of the comment is, that title and aim of the study should correspond, our answer is, that this is the case, because at both places we mention the validation of an instrument to assess the quality of information.

*2- In the abstract, the methods section needs to be revised to include the necessary information about the research methodology, participants, and research instruments.*

**Response:** The manuscript reports on five (two qualitative and three quantitative) separate studies. It is not possible to provide all details to each of the studies in the abstract. The number of participants is provided for the quantitative studies, while the qualitative studies anyway would work based on the saturation principle. All those details are provided in the manuscript.

*3- The background section is so brief and the gap in the existing knowledge needs to be addressed.*

**Response:** The background section is short because we refer to the protocol. We do not see a need to plagiarize our own paper, which is openly accessible. However, the gap regarding existing knowledge is clearly defined and justified in the current manuscript.

Our systematic literature review identified 27 assessment tools, however none of them fully fulfils the requirements to include evidence-based criteria nor can be used by untrained lay people.

*4- In the methods section, the number of the participants and experts in each substudy needs to be added.*

**Response:** In each substudy, the number of participants, experts and applicable materials is indicated.

*5- In the methods and results sections, the four steps of instrument development, namely; concept identification; item construction; validity testing and reliability testing or any other frameworks needs to be followed.*

*Davis AE. Instrument development: getting started. J Neurosci Nurs. 1996 Jun;28(3):204-7.*

**Response:** Our development is documented in various studies building up on each other. The guideline development is published in an own paper refereeing to other papers founding the concept of evidence-based health information. The item construction is described within the draft of the instrument in a previous paper. The current paper is describing the previously planned steps of first reliability and then validity testing. In the reviewer’s comment we do not find a justification for the need to restructure the methods chapter or to refer to a framework as these steps follow common standards.

*6- The discussion section needs to be supported by the relevant or similar studies.*

*7- The number of references is limited.*

**Response:** We added a couple of reviews underpinning our statement regarding the lacking focus on the availability of evidence-based information. However, we cannot believe that this criticism has led to the decision to reject the manuscript.

I am, therefore, taking the liberty of re-submitting this manuscript because we are convinced that we have prepared a high quality and interesting paper, which we would like very much to see published in your esteemed journal.

Yours sincerely,

Jürgen Kasper, Prof. Dr. phil.

OsloMet Metropolitan University, Oslo, Norway
